# Nanostructure-Integrated Electrode Based on Ni/NiO Coaxial Bilayer Nanotube Array with Large Specific Capacitance for Miniaturized Applications

**DOI:** 10.3390/ma18061286

**Published:** 2025-03-14

**Authors:** Qianxun Gong, Xiaoyan Huang, Yong Liu, Sijie Zhang, Tian Yu

**Affiliations:** 1 College of Physics, Sichuan University, Chengdu 610065, China; suoketuo@163.com (Q.G.); huangxiaoyan_scu@163.com (X.H.); sijie.zhang@scu.edu.cn (S.Z.); 2 Analytical & Testing Center, Sichuan University, Chengdu 610065, China; liuyong_ly@sina.com; 3 School of Science, Guizhou University of Engineering Science, Bijie 551700, China

**Keywords:** coaxial bilayer nanotube array, integrated electrode, supercapacitor, Ni/NiO

## Abstract

The fast development of portable electronics demands electrodes for supercapacitors that are compatible with miniaturized device applications. In this study, an orderly aligned coaxial bilayer nanotube array made of transition metal/transition metal oxides was adopted as a nanostructure-integrated electrode for applications as miniaturized micro-supercapacitors. Using Ni and NiO as our model materials, the corresponding Ni/NiO-CBNTA electrodes were fabricated using templated growth and post-thermal oxidation. The Ni shells served as parts of the 3D nano-architectured collector, providing a large specific surface area, and the pseudocapacitive NiO layers were directly attached and electrically connected to the collector without any additives. The vertical growth of orderly aligned Ni/NiO-CBNTAs successfully avoided the underutilization of capacitive nanomaterials and allowed the electrolyte to be fully accessed, which manifested full charge storage capabilities under the miniaturizing. It was demonstrated that Ni/NiO-CBNTAs can serve as miniaturized electrodes with an improved specific capacitance of 1125 F/g ≅ 3 A/g, which is comparable to that obtained in a massive load electrode prepared by the conventional slurry-coating technique.

## 1. Introduction

In recent decades, the growing demand for renewable energy systems has driven extensive research on high-performance energy storage devices, among which supercapacitors (SCs) have emerged as one of the most promising technologies, owing to their unique combination of high power density, rapid charge–discharge capability, and long-term cycling stability [1,2]. Extensive studies have shown that the performance of SCs is fundamentally determined by their electrode materials, making the development of advanced electrode architectures a central focus in this field [3,4]. Particularly for miniaturized applications such as on-chip micro-supercapacitors (MSCs) in Internet of Things (IoT) devices, electrode design must address challenges arising not only from planarizing and miniaturizing but also from the significant deterioration of charge storage performance as SCs scale down [5,6,7,8]. This performance degradation compared to conventional SCs is easy to understand: in conventional SCs, excessive capacitive nanomaterials can overcome the ill connection and underutilization caused by random assembly between capacitive nanomaterials and collectors. However, the miniaturization of SCs substantially limits the accommodable amount of the capacitive nanomaterials.

Herein, we show that aligned coaxial bilayer nanotube arrays (CBNTAs) based on metallic Ni and its oxide NiO can be explored as the desired electrode for SCs in miniaturized applications. Transition metal oxides are widely used as supercapacitor electrode materials [9,10,11], including NiO [11,12], MnO_x_ [13,14], Co_3_O_4_ [15,16], and RuO_2_ [17,18]. Among them, NiO holds great promise due to its abundance of raw materials, low cost, environmental friendliness, and relatively large theoretical specific capacity [9,11,12]. In our Ni/NiO-CBNTA design, the highly conductive metallic Ni shells grow vertically aligned and are connected into a network through a bottom metal layer. These Ni tubular shells, together with the bottom layer, serve as a three-dimensional (3D) nano-architectured collector in the electrode. Since this 3D nano-architectured collector increases the porosity, enlarges the specific surface area of the collector, and accommodates more capacitive nanomaterials, it helps fulfill the miniaturized applications of the SCs. The coaxial NiO tubular layers, functioning as the capacitive nanomaterials, grow natively from the Ni shells and ensure intimate interfacial contact with the 3D nano-architectured collector, thus avoiding undesirable overlapping and advances the utilization of the capacitive nanomaterials. In addition, as the coaxial NiO layers grow natively from the Ni shells, a direct and robust electrical connection between the capacitive nanomaterials and the 3D nano-architectured collector is also expected. Furthermore, as all the Ni/NiO coaxial bilayer nanotubes are grown vertically and aligned, their hollow structures provide organized channels for the electrolyte to enter the interior of the electrodes. Therefore, the Ni/NiO-CBNTAs as an integrated design of both capacitive nanomaterials and 3D nano-architectured collector provide nanostructure-integrated electrodes which can manifest full charge storage capabilities when miniaturized and fulfill the miniaturized applications of the SCs.

## 2. Materials and Methods

**Figure 1:**
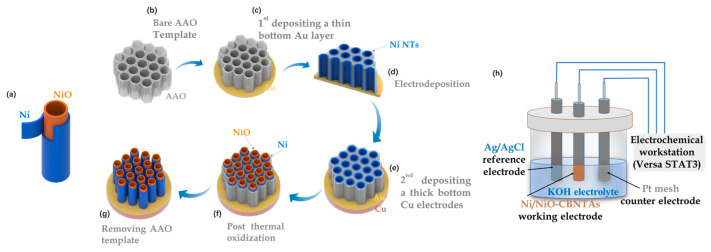
(**a**) Diagram of a single Ni/NiO coaxial bilayer nanotube. (**b**–**g**) Sketch to illustrate the preparation of the nanostructure-integrated electrode based on Ni/NiO-CBNTAs. (**h**) Diagram of the three-electrode cell.

The Ni/NiO-CBNTAs were prepared by combining template-assisted electrodeposition with post-thermal oxidization, as illustrated in Figure 1a–g. This templated growth of CBNTAs by electrodeposition is a well-established low-cost technique [19,20]. In our experiments, highly ordered porous anodic aluminum oxide (AAO) with an average pore diameter of $d_{AAO}=300\;\mathrm{nm}$ was used as the template. An Au seed layer of 75 nm in thickness was evaporated on the bottom of the AAO template, which also served as the working electrode during the electrodeposition of the Ni nanotubes. The electrodeposition was conducted at room temperature using an electrochemical workstation (Versa STAT3, AMETEK Scientific Instruments, Oak Ridge, TN, USA), where a constant potential of $E_{dep.}=-1.0\;V$ (vs the saturated calomel reference electrode) was applied, and an aqueous solution of NiSO_4_ 0.038 M was used as the electrolyte. The electrodeposition time $t_{dep.}=3000\;s$ was chosen based on our previous results to control the length of the nanotubes [20]. Post-thermal oxidization was carried out in air atmosphere using a tubular oven. A series of oxidization temperatures were tried: $T_{O}=200\;{}^{\circ}C$, 300 °C, 400 °C, and 500 °C. The heating/quenching rate and the oxidization time were fixed at 10 °C/min and 5 °C/min, respectively. After the oxidization, a thick Cu layer of 1 μm was grown onto the Au seed layer, which firmly connected all nanotubes into a conductive network and, together with the Ni shells, served as the 3D nano-architectured collector. Finally, the template was removed by selective etching in a 1 M KOH aqueous solution. As a comparison, electrodes based on Ni/NiO coaxial bilayer nanowire arrays were prepared similarly, in which the Au seed layer was changed to 300 nm.

The morphology and chemical composition of the Ni/NiO-CBNTAs were examined by scanning and transmission electron microscopy (SEM JSM-7500F, JEOL, Tokyo, Japan and TEM Tecnai G2 F20 S-TWIN, FEI, Hillsboro, OR, USA), energy-dispersive spectroscopy (EDS, INCA X-Max50, Oxford Instrument, Abingdon, UK), and Raman spectrum (LabRAM Soleil, HORIBA FRANCE SAS, Palaiseau, France). Noticing that Ni and NiO were distinguishable magnetic materials, ferromagnetic and antiferromagnetic, respectively, the magnetic properties of the Ni/NiO-CBNTAs were investigated using a vibrating sample magnetometer (PPMS-VSM system, Quantum Design, San Diego, CA, USA), which uniquely indicated the composition and structure of the Ni/NiO-CBNTA-based nanostructure-integrated electrodes prepared under different $T_{O}$. The room-temperature electrochemical performance, including cyclic voltammetry (CV) and galvanostatic charge/discharge (GCD), were characterized using the electrochemical workstation and a three-electrode cell (as shown in Figure 1h), where Pt mesh, Ag/AgCl electrode, and Ni/NiO-CBNTAs served as counter, reference, and working electrodes, respectively. Analytical-grade KOH was dissolved in deionized water (1M) and used as the electrolyte.

## 3. Results and Discussion

**Figure 2:**
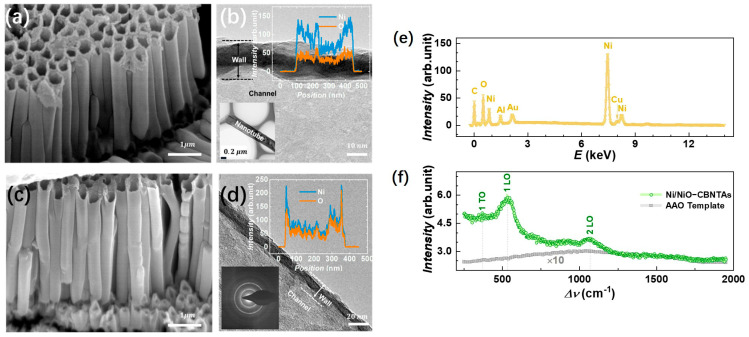
(**a**,**b**) shows the SEM and TEM images for the raw Ni nanotube array, respectively. The insert of (**b**) shows the element profile of the raw Ni nanotube. (**c**,**d**) shows the SEM and TEM images for the Ni/NiO-CBNTAs obtained after oxidation in air at $T_{O}=500\;{}^{\circ}C$. The insert suggests that the atomic ratio of Ni to O is almost 1:1, and clear electron diffraction rings are observed after oxidization. (**e**) EDS for the Ni/NiO-CBNTA nanostructure-integrated electrodes. (**f**) Raman spectrum for Ni/NiO-CBNTAs (green) and the bare AAO template (gray).

Figure 2a,c shows a typical electron microscopy image for the raw Ni nanotube array and the Ni/NiO-CBNTAs, respectively. It can be seen that they were similar in morphology. Both were 3D nanostructures constituted by orderly aligned and vertically grown individual nanotubes. The hollow structures of these nanotubes were indicated by their top-open holes and were further confirmed from the TEM results. As shown in Figure 2b,d, the brighter contrast compared to that of the nanotube walls clearly indicated the channels of the nanotubes. As expected, the outer diameter of the nanotubes was around 300 nm, which was consistent with the pore size of the template. The nanotube wall thickness and axial length of the nanotubes estimated from the above electron microscopy were 50 nm and 2 μm, respectively. The channels were favorable for our nanostructure-integrated electrodes, as they offered pathways for the electrolyte to enter the interior of the electrodes. Keeping the morphology unaltered, the thermal oxidization modified the composition of these CBNTAs. As indicated by the elemental-section profiles, shown as the inserts of Figure 2b,d, a small amount of oxygen was found in the raw Ni nanotubes, while the atomic ratio of O/Ni almost reached 1 after oxidization under $T_{O}=500\;{}^{\circ}C$ for 30 min. Oxygen was found in the raw Ni nanotube because the natural oxidation process started immediately after deposition [21,22]. Clear electron diffraction rings were absent in the raw Ni nanotube arrays but appeared after the thermal oxidization, indicating a crystal structure transformation accompanying the composition modification.

In the EDS measurement, Cu and Au were the main metal elements found in addition to Ni in the nanostructure-integrated electrodes, as shown in Figure 2e. Conventionally, a thin layer of NiO can be identified by using X-ray diffraction (XRD). However, the peaks in the XRD corresponding to either NiO or Ni were not observed for these nanotube arrays. This was partly because the nanotube only had a thin wall with very limited materials and also because the hollow structure of the nanotube arrays captured and diffusively reflected the incident photons. The growth of the NiO coaxial layers from raw Ni was further characterized by Raman spectroscopy. The Raman spectroscopy showed higher sensitivity because it collected focused scattering contributions arising locally and predominantly from the surface [23]. As shown in Figure 2f, a series of Raman shift peaks corresponding to stretches of Ni-O bonds were observed in a wavenumber (Δν) ranging from 200–$2000{\;\mathrm{cm}}^{-1}$. It was recognized that the Raman bands located at $\Delta \nu =360\;{\mathrm{cm}}^{-1}$ and $530\;{\mathrm{cm}}^{-1}$ were one-phonon first-order transverse optical (1P-TO) and longitudinal optical (1P-LO) modes of NiO, respectively, while the band at $\Delta \nu =1053{\;\mathrm{cm}}^{-1}$ was due to the two-phonon second-order longitudinal optical (2 LO) mode [21,24,25]. Figure 2f also presents the Raman spectrum for the bare AAO template, where only a weak broad background of amorphous Al_2_O_3_ was found.

**Figure 3:**
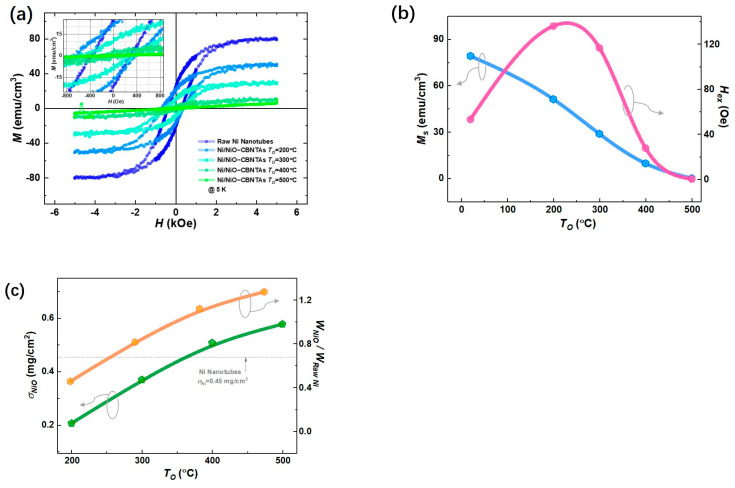
Magnetic properties of the raw Ni nanotube array and the nanostructure-integrated electrodes prepared at different $T_{O}$. (**a**) Magnetic hysteresis (*M-H*) loops measured at 5 K. (**b**) Specific saturation magnetization ($M_{s}$) (blue) and exchange bias field ($H_{ex}$) (pink). (**c**) The mass load of NiO ($\sigma_{NiO}$) for Ni/NiO-CBNTAs prepared (green) and the mass ratio between NiO and raw Ni ($W_{NiO}/W_{Raw\;Ni}$) (orange) under different $T_{O}$.

Figure 3a summarizes magnetic hysteresis (M−H) loops measured at 5 K after magnetic field cooling (FC) for both the raw Ni nanotube array and the nanostructure-integrated electrodes. Considering that Ni is a ferromagnetic metal, whereas NiO is an antiferromagnet [26], these center-shifted ferromagnetic hysteresis loops (see insert of Figure 3a) suggest that the raw Ni layer was only partially oxidized into NiO after post-thermal oxidization under $T_{O}=200\;{}^{\circ}C$, 300 °C, and 400 °C. This shift is a feature of the exchange bias (EB) effect in ferromagnet/antiferromagnet systems and evidenced the formation of the Ni/NiO interfaces in the nanostructure-integrated electrodes [27]. Compared with the specific saturation magnetization ($M_{s}$) for bulk Ni ($668.3\;\mathrm{emu}/{\mathrm{cm}}^{3}@\;5\;K$) [20], a small magnetization ($79.6\;\mathrm{emu}/{\mathrm{cm}}^{3\;}@\;5\;K$) was obtained for the raw Ni nanotubes, which was reasonable, as the nanotubes involved plenty of hollows unfilled by Ni. As seen in Figure 3b, the $M_{S}$ decreased with increasing $T_{O}$. As NiO is an antiferromagnet showing no net magnetization, this is consistent with the expectation that raising $T_{O}$ promotes the growth of NiO and thus consumes more Ni. It was also noticed in Figure 3b that the exchange bias field ($H_{ex}$) vanished at $T_{O}=500\;{}^{\circ}C$, suggesting that all Ni was converted into NiO.

In addition, the difference in magnetic properties between the raw Ni nanotube array and the Ni/NiO-CBNTA electrodes provided a unique estimation of the amount of NiO. For a given electrode, its magnetic moment was determined by the amount of Ni, which decreased after the post-thermal oxidation due to the conversion into the NiO layer. Therefore, the ratio of Ni converted into NiO can be evaluated by the changes in magnetization using Equation (1):

(1) $$\gamma_{Ni\to NiO}=\frac{\Delta M_{s}}{M_{s}}=\frac{M_{s}-M_{s}^{'}}{M_{s}}$$

Thus, the mass weight of the NiO ($W_{NiO}$) in the final Ni/NiO-CBNTA electrodes can be calculated using Equation (2):

(2) $$W_{NiO}=W_{Ni}\gamma_{Ni\to NiO}\frac{\mu_{NiO}}{\mu_{Ni}}$$

where $\mu_{NiO}$, $\mu_{Ni}$, $M_{S}$, $M_{S}^{'}$, and $W_{Ni}$ are the molecular weights of NiO and Ni, the specific saturated magnetization measured before and after post-thermal oxidation, and the mass of the raw Ni, respectively. As summarized in Figure 3c, using $\sigma_{Ni}=0.45\;\mathrm{mg}/{\mathrm{cm}}^{2}$, the area density of the raw Ni nanotubes obtained from the ICP measurement, the mass loads of NiO were determined as $0.206\;\mathrm{mg}/{\mathrm{cm}}^{2}$, $0.369\;\mathrm{mg}/{\mathrm{cm}}^{2}$, $0.507\;\mathrm{mg}/{\mathrm{cm}}^{2}$, and $0.577\;\mathrm{mg}/{\mathrm{cm}}^{2}$ for the nanostructure-integrated electrodes prepared under $T_{O}=200\;{}^{\circ}C$, 300 °C, 400 °C, and 500 °C, respectively.

**Figure 4:**
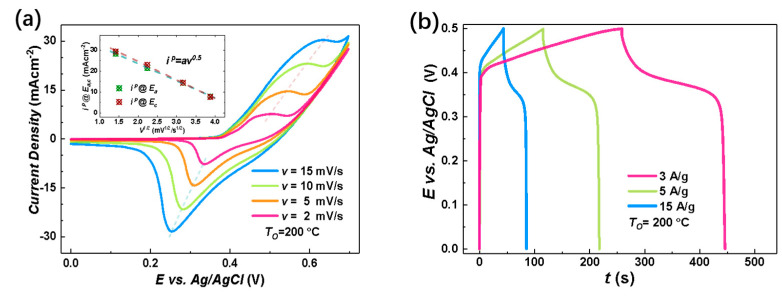

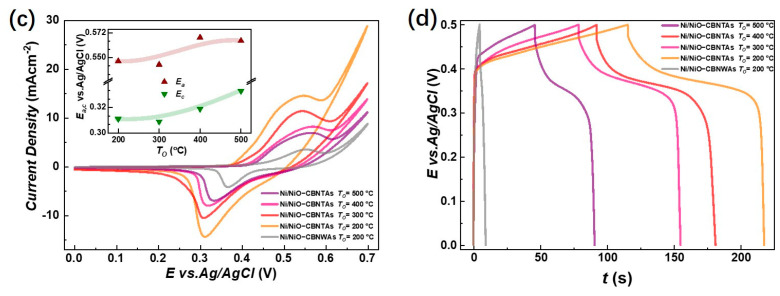
Electrochemical charge storage properties of Ni/NiO-CBNTA nanostructure-integrated electrodes. (**a**) Cyclic voltammetry (CV) curves of the nanostructure-integrated electrodes prepared at different scanning rates (υ). The insert of (**a**) shows the oxidation peak (wine) and redox peak (green) current densities as a function of scan rate, and (**b**) shows the galvanostatic charge–discharge (GDC) measured at a series of current densities of 3 A/g, 5 A/g, and 15 A/g. (**c**) CV curves at the scan rate of 5 mV/s for the Ni/NiO-CBNTA electrodes prepared under different $T_{O}$ and the electrode based on the Ni/NiO coaxial bilayer nanowire array. The insert of (**c**) shows the dependence of oxidation peak (wine) and redox peak (green) current densities on the $T_{O}$. (**d**) GDC measured under the same mass current density of 5 A/g for Ni/NiO-CBNTA electrodes prepared under different $T_{O}$ and the electrode based on the Ni/NiO coaxial bilayer nanowire array.

The electrochemical charge storage properties of the nanostructure-integrated electrodes were characterized by CV measurements at room temperature in the potential (E) range of 0−0.7 V vs Ag/AgCl using 1 M KOH aqueous solution as the electrolyte. Figure 4a shows an example of the CV curves for the nanostructure-integrated electrode prepared under $T_{O}=200\;{}^{\circ}C$ at various scan rates (υ). In an alkaline solution, both the electric double layer (EDL) at the electrode/electrolyte interface and the Faradaic redox reactions may contribute to the total capacitance for capacitive metal oxides. The EDL usually results in a rectangular CV curve with no distinct peak. Therefore, the peaks around 0.25–0.3 V and 0.45–0.6 V in Figure 4a clearly excluded the EDL as the dominant charge storage mechanism in our Ni/NiO-CBNTA nanostructure-integrated electrodes and suggests that the charge storage mainly relied on mutual transformations between Ni(Ⅱ) and Ni(Ⅲ) [9,10,11,12], according to Equation (3):

(3) $$NiO+zOH^{-}⇌zNiOOH+\left(1-z\right)NiO+ze^{-}$$

According to this Faradic redox reaction, the observed peaks were assigned as the anodic peak at $0.45\;V<E_{a}<0.6\;V$, due to the oxidation of NiO into NiOOH, and the cathodic peak at $0.25\;V<E_{c}<0.3\;V$ for the reverse process [21,28,29]. From Figure 4a, it can be seen that as we increased the υ, the area enclosed by the CV curves was enlarged without distortion in shape, revealing the reasonable kinetic reversibility of the Ni/NiO-CBNTA nanostructure-integrated electrode. It is also noticeable in Figure 4a that as the υ increased, the anodic and cathodic peaks shifted in positive and negative directions, respectively. This caused an increase in the potential difference between the anodic peak and the cathodic peak, $\Delta E=E_{a}-E_{c}$, which was commonly observed and attributed to the limitation of the ion diffusion rate [29]. This ion diffusion-controlled capacitance was further indicated by the exponent (b) in the relationship between the v and the peak redox current density ($i_{p}=i\;@\;E_{a,c}$), as shown with Equation (4):

(4) $$i_{p}=av^{b}$$

where a is a proportionality constant. As shown in the inset of Figure 4a, a linear relation was found with b=0.5, indicating that the charge storage was controlled by the intercalation/de-intercalation of $OH^{-}$ in the crystal framework of NiO [30].

To further evaluate the charge storage performances of the Ni/NiO-CBNTA nanostructure-integrated electrode, GDC measurements were carried out between working potential 0 and 0.5 V vs. Ag/AgCl and presented in Figure 4b. The non-linear E–t responses and plateaus in the discharge branches again indicated the occurrence of Faradaic redox reactions in our Ni/NiO-CBNTA nanostructure-integrated electrode. It can be seen that all discharge branches were divided into two sections: a resistive component arising from the sudden voltage drops representing the voltage change due to the internal resistance and a capacitive component related to the voltage change due to the change in energy within the capacitance [31]. From the GDC, the specific capacitance ($C_{m}$) was calculated using Equation (5):

(5) $$C_{m}=\frac{It}{W_{NiO}\Delta V}$$

where I is the applied current, t is the discharging time, $W_{NiO}$ is the mass of active materials, and ΔV is the working potential. For the mass current density of 3 A/g, 5 A/g, and 15 A/g, the obtained $C_{s}$ were 1125 F/g, 1040 F/g, and 651 F/g, respectively. The charge storage performance for the Ni/NiO-CBNTA nanostructure-integrated electrodes prepared under different $T_{O}$ were also investigated. As shown in Figure 4c, the enlarging of the enclosed area suggests that Ni/NiO-CBNTA nanostructure-integrated electrodes prepared under a lower $T_{O}$ had better electrochemical charge storage performance. This dependence of $C_{m}$ on $T_{O}$ was also verified in the GCD measurements. As shown in Figure 4d, a longer discharge time and more distinct plateau regions were observed for the Ni/NiO-CBNTA nanostructure-integrated electrodes prepared under a lower $T_{O}$.

**Figure 5:**
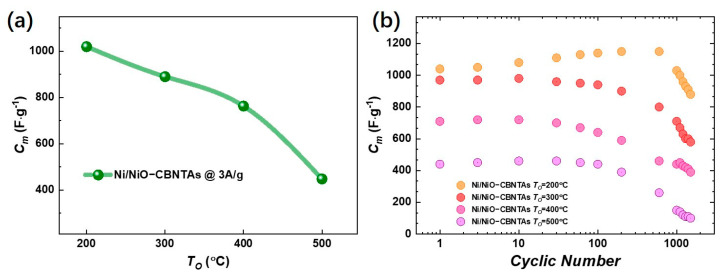
(**a**) Summary of the dependence of the specific capacitance ($C_{m}$) on the post-thermal oxidization temperature $T_{O}$ at a current density of 3 A/g. (**b**) The cyclic GCD results for Ni/NiO-CBNTA nanostructure-integrated electrodes at a current density of 3 A/g.

Figure 5a summarizes $C_{m}$ for the electrodes prepared under different $T_{a}$, and the $C_{m}$ evaluated for $T_{O}$ = 300 °C, 400 °C, and 500 °C were 890 F/g, 763 F/g, and 448 F/g at 3 A/g, respectively. It was conceivable that increasing $T_{O}$ had multiple effects. Firstly, it added the amount of the pseudocapacitive NiO by promoting oxidation, which facilitated charge storage performance. Meanwhile, the growth of NiO natively from the raw metallic shells consumed the conductive Ni in the nanostructured collector, which unavoidably increased the resistivity. It was the competition between them that determined the dependence of charge storage performance on the $T_{O}$. Noticing that the enlargement of the enclosed area in CV curves at the same scan rate was mainly due to increasing the redox current density, similar to lowering $T_{O}$, and though the $E_{a,c}$ were shifted but were shifted both positively, keeping the $\Delta E=E_{a}-E_{c}$ almost unaltered, we ascribed the deterioration of charge storage performance as it increased to the aggressive growth of the NiO layer. As the depth of intercalation/de-intercalation of ions was restricted to the outermost surface of the NiO, the aggressive growth of the NiO layer had limited contribution to the Faradic redox reactions but counterproductively increased resistivity [32,33].

The cyclic stability was tested by cycling the GCD with a mass current density of 3 A/g. It can be seen from Figure 5b that the $C_{m}$ gradually decreased from 1125 F/g to 890 F/g for the Ni/NiO-CBNTA nanostructure-integrated electrodes prepared under $T_{O}=200\;{}^{\circ}C$, giving a capacitance retention of 80%. It was noticed that the capacitance retention also increased with lowering $T_{O}$. The decline of $C_{m}$ in metal oxides-based capacitive nanomaterials is usually attributed to inadequate de-intercalation of ions [34,35]. Under a lower $T_{O}$, the thinner NiO thickness advanced the de-intercalation of ions and thus enhanced capacitance retention.

**Figure 6:**
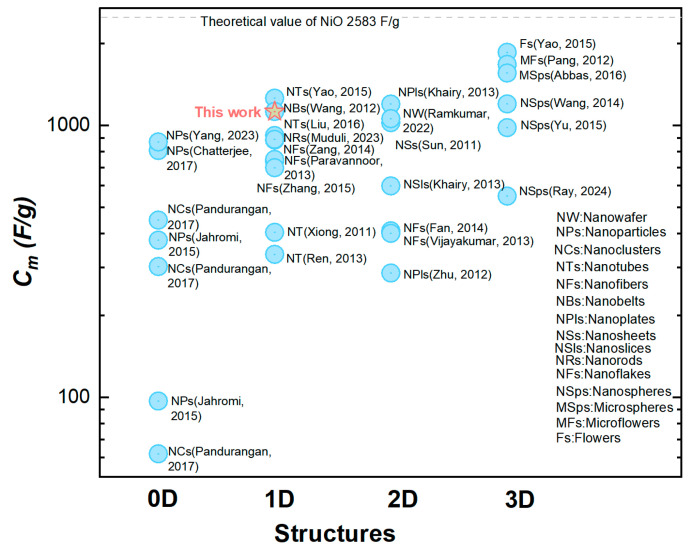
A comparison of the specific capacitance ($C_{m}$) in NiO-based electrodes with different nanostructures [24,30,36,39,40,41,42,43,44,45,46,47,48,49,50,51,52,53,54,55,56,57,58,59,60].

This relatively large $C_{m}=1125\;F/g$ was mainly attributed to making the most utilization of the capacitive nanomaterials NiO in our orderly organized coaxial nanotube array structure. This structural advantage was further demonstrated by the comparison between two electrodes constituted by the CBNTAs and the coaxial bilayer nanowire arrays (CBNWAs), respectively. As presented in Figure 4c,d, though these two electrodes were prepared under similar procedures and were both constituted by one-dimensional components of the same outer diameter and axial length, they showed distinguishable charge storage performances. The electrode based on nanowire arrays only exhibited a smaller redox current density response in the CV measurement (see Figure 4c) and shorter discharging time in the GCD characterization (see Figure 4d), indicating an inferior change storage performance, compared with that of the electrode based on the CBNTAs. Further support for the NiO/Ni-CBNTAs’ ability to achieve a large $C_{m}$ came from electrochemical impedance spectroscopy (EIS) measurements (see Supplementary Materials). The NiO/Ni-CBNTAs exhibited a significantly lower charge-transfer resistance $R_{ct}$ than the NiO/Ni-CBNWAs. Since $R_{ct}$ usually arises from the ionic resistance of the electrolyte, the intrinsic resistance of active materials, and contact resistance at the active material/current collector interface, this reduction in $R_{ct}$ in the NiO/Ni-CBNTAs suggested enhanced ionic accessibility and improved charge transfer kinetics. Finally, we note that while further optimization and electrochemical dynamics analyses are required for large-scale device applications and to understand the influence of growth parameters on $C_{m}$ [36,37,38], our lab-scale results demonstrated the great potential of Ni/NiO-CBNTAs-based nanostructure-integrated electrodes to achieve large $C_{m}$. This can also be seen from Figure 6, where the $C_{m}$ for the NiO-based electrode of various nanostructures are summarized.

## 4. Conclusions

Electrodes compatible with miniaturized applications were prepared based on arrays of vertically aligned Ni/NiO coaxial bilayer nanotube arrays by low-cost templated growth and post-thermal oxidization, in which the inner NiO layer was automatically integrated with the metallic outer Ni layer, forming a nanostructure-integrated electrode. In this Ni/NiO-CBNTA nanostructure-integrated electrode, the Ni shells served as parts of the 3D nano-architectured collector, providing a large specific surface area. The pseudocapacitive NiO layers were directly attached and electrically connected to the collector without any additives. Their orderly organized hollow structure further urged electrolyte to enter the interior of the electrodes. A specific capacitance of 1125 F/g was achieved at 3 A/g, which is comparable to that obtained in electrodes with a massive load of the capacitive nanomaterials. The charge-storage performance of the electrode was found to be sensitive to its preparation condition. The specific capacitance decreased as the post-thermal oxidization temperature increased, which was understood to be based on the balance between the oxidation growth of NiO by consuming conductive Ni and the limitation of intercalation/de-intercalation of ions, as well as the resistivity of the electrodes.

## Data Availability

The original contributions presented in this study are included in the article. Further inquiries can be directed to the corresponding author.

## Supplementary Materials

The following supporting information can be downloaded at: https://www.mdpi.com/article/10.3390/ma18061286/s1, Figure S1: Nyquist plot of EIS for NiO/Ni-CBNTAs and NiO/Ni-CBNWAs. References [36,37,38] are cited in the Supplementary Materials.

## Abbreviations

The following abbreviations are used in this manuscript:

SC	Supercapacitors
IoT	Internet of Things
MSC	Micro-supercapacitor
CBNTA	Coaxial Bilayer Nanotube Array
TM	Transition Metal
TMO	Transition Metal Oxide
AAO	Anodic Aluminum Oxide
GCD	Galvanostatic Charge/Discharge
1P-TO	First-Order Transverse Optical
1P-LO	First-Order Longitudinal Optical
EDL	Electric Double Layer
CBNWA	Coaxial Bilayer Nanowire array
CV	Cyclic Voltammetry
EIS	Electrochemical Impedance Spectroscopy
