# Supplementary material for: Nanostructure-Integrated Electrode Based on Ni/NiO Coaxial Bilayer Nanotube Array with Large Specific Capacitance for Miniaturized Applications
Source: Materials (Basel). 2025 Mar 14;18(6):1286. doi: 10.3390/ma18061286 (PMC11944225; doi:10.3390/ma18061286)
Supplement: Supplementary file 1 [file materials-18-01286-s001.zip › materials-3480862-supplementary.pdf]

Supplementary Materials

Electrochemical impedance spectroscopy (EIS) comparison between the NiO/Ni-CBNTAs and NiO/Ni-CBNWAs (prepared at $T_O = 200$ ℃)

The EIS was investigated by applying the frequency from 100 kHz to 0.1 Hz with 10 mV of ac amplitude using an electrochemical workstation (Versa STAT3). As seen in Fig.S1, the impedance spectra of the NiO/Ni-CBNTAs and the NiO/Ni-CBNWAs are almost similar in shape, consisting of one semicircle at high frequency followed by a straight line in the low-frequency range.

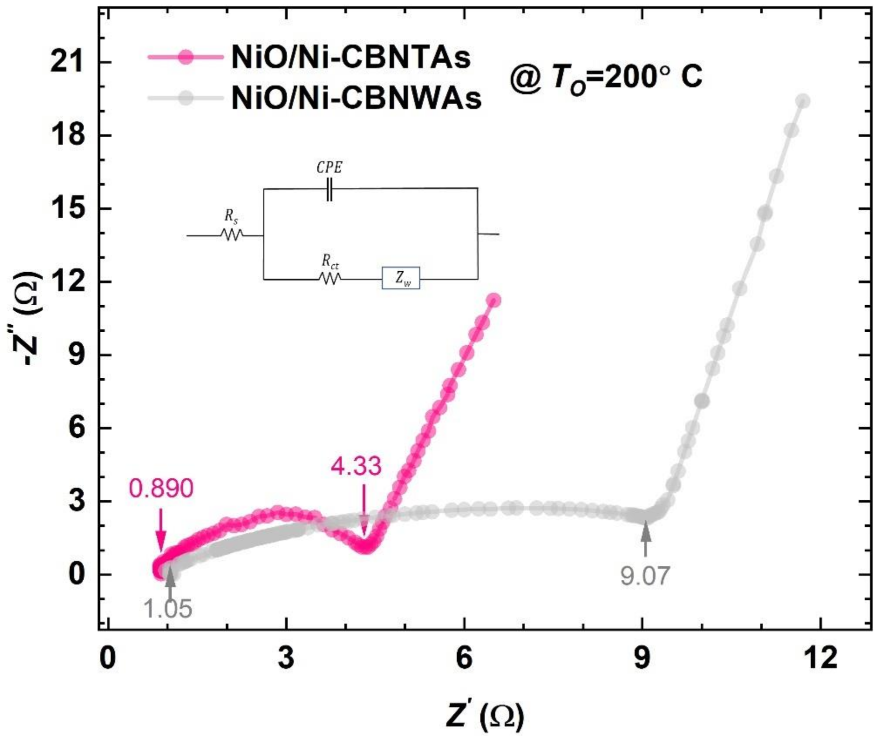

Fig. S1 Nyquist plot of EIS for NiO/Ni-CBNTAs and NiO/Ni-CBNWAs

The equivalent circuit corresponding to the Nyquist plot is presented in the inset of Fig. S1, where R_s is the solution resistance of the electrochemical system, CPE is the so-called constant phase element, W_z is the Warburg impedance (resulting from the frequency dependence of ion diffusion/transport in the electrolyte), and R_{ct} is Faradaic interfacial charge-transfer resistance [S1-S3]. It can be seen from Fig. S1 that, in the low-frequency

region, their solution resistance R_s are nearly the same for both the NiO/Ni-CBNTAs and the NiO/Ni-CBNWAs. However, in the high-frequency region, the charge-transfer resistances show a significant difference. The R_{ct} estimated from the diameter of these semicircles for NiO/Ni-CBNTAs and NiO/Ni-CBNWAs are $3.44\,\Omega$ and $8.02\,\Omega$ respectively. Since the R_{ct} normally results from the ionic resistance of electrolyte, the intrinsic resistance of the active materials, and the contact resistance between the active material and current collector, the lower R_{ct} in NiO/Ni-CBNTAs suggests that the coaxial bilayer nanotube array structure enhances ionic accessibility as well as charge transfer at the electrode-electrolyte interface, thereby favoring the attainment of a large C_m.

References：

S1. Lazanas, A. Ch.; and Prodromidis, M. I. Electrochemical Impedance Spectroscopy—A Tutorial, *ACS Measurement Science Au* **2023**, 3(3) 162–193.

S2. Ren, B.; Fan, M.; Liu, Q.; Wang, L.; Song, D.; Bai, X. Hollow NiO nanofibers modified by citric acid and the performances as supercapacitor electrode, *Electrochimica Acta* **2013**, 92 197– 204.

S3. Sutar, S. H.; Patil, S. B.; Bansal, L.; Sadale, S. B.; Kumar, R.; Mujawar, S. H. Electrochemical and impedance analysis of nickel oxide nanoflakes-based electrodes for efficient chromo supercapacitors *Electrochimica Acta* **2024**, 498 144614.
